# Very Early Passive Cycling Exercise in Mechanically Ventilated Critically Ill Patients: Physiological and Safety Aspects - A Case Series

**DOI:** 10.1371/journal.pone.0074182

**Published:** 2013-09-09

**Authors:** Ruy Camargo Pires-Neto, Yurika Maria Fogaça Kawaguchi, Adriana Sayuri Hirota, Carolina Fu, Clarice Tanaka, Pedro Caruso, Marcelo Park, Carlos Roberto Ribeiro Carvalho

**Affiliations:** 1 Physical Therapy Service, Instituto Central do Hospital das Clínicas da Faculdade de Medicina da Universidade de São Paulo – São Paulo (ICHC-FMUSP), São Paulo, Brazil; 2 Department of Pathology, Faculdade de Medicina da Universidade de São Paulo (FMUSP), São Paulo, São Paulo, Brazil; 3 Department of Physical Therapy, Speech Pathologist Therapist and Occupational Therapy, Faculdade de Medicina da Universidade de São Paulo (FMUSP), São Paulo, São Paulo, Brazil; 4 Division of Pulmonary and Critical Care, Heart Institute (InCor), Hospital das Clinicas da Faculdade de Medicina da Universidade de São Paulo, São Paulo, São Paulo, Brazil; 5 Medical Emergency ICU - Hospital das Clínicas da Faculdade de Medicina da Universidade de São Paulo (ICHC/FMUSP), São Paulo, São Paulo, Brazil; Universidad Europea de Madrid, Spain

## Abstract

**Introduction:**

Early mobilization can be performed in critically ill patients and improves outcomes. A daily cycling exercise started from day 5 after ICU admission is feasible and can enhance functional capacity after hospital discharge. In the present study we verified the physiological changes and safety of an earlier cycling intervention (< 72 hrs of mechanical ventilation) in critical ill patients.

**Methods:**

Nineteen hemodynamically stable and deeply sedated patients within the first 72 hrs of mechanical ventilation were enrolled in a single 20 minute passive leg cycling exercise using an electric cycle ergometer. A minute-by-minute evaluation of hemodynamic, respiratory and metabolic variables was undertaken before, during and after the exercise. Analyzed variables included the following: cardiac output, systemic vascular resistance, central venous blood oxygen saturation, respiratory rate and tidal volume, oxygen consumption, carbon dioxide production and blood lactate levels.

**Results:**

We enrolled 19 patients (42% male, age 55±17 years, SOFA = 6 ± 3, SAPS3 score = 58 ± 13, PaO_2_/FIO_2_ = 223±75). The median time of mechanical ventilation was 1 day (02), and 68% (n=13) of our patients required norepinephrine (maximum concentration = 0.47 µg.kg ^-1^.min^-1^). There were no clinically relevant changes in any of the analyzed variables during the exercise, and two minor adverse events unrelated to hemodynamic instability were observed.

**Conclusions:**

In our study, this very early passive cycling exercise in sedated, critically ill, mechanically ventilated patients was considered safe and was not associated with significant alterations in hemodynamic, respiratory or metabolic variables even in those requiring vasoactive agents.

## Introduction

Recent studies have shown that early physical mobilization can be performed in critically ill patients even when these patients require mechanical ventilation [1–4]. Early mobilization is feasible and safe [1]. It is also associated with a decrease in hospital and intensive care unit (ICU) length of stay [3], better functional outcomes at hospital discharge, shorter duration of delirium and an increase in days free of mechanical ventilation [4]. Moreover, if an early mobility intervention is not undertaken in the ICU, the rates of hospital readmission and death during the first year after hospital discharge increase [5].

It has been shown that a daily cycling exercise (passive and active) can enhance the functional capacity, self-perceived functional status and quadriceps muscle strength of ICU patients [6]. However, in that study, patients were screened for exercise five days after ICU admission, and the mean interval at which the treatment group began exercising was 14 days [6].

Because early mobilization is associated with better clinical outcomes for ICU patients, we hypothesized that an earlier intervention might further improve clinical outcomes. Before addressing this question, it is important to evaluate the safety of a cycling exercise performed earlier than previously evaluated [6]. Therefore the objective of this study was to evaluate the hemodynamic, respiratory and metabolic effects of a cycling exercise performed during the first 72 hrs of mechanical ventilation.

## Methods

The study was approved by the local ethics committee (Comissão de Ética para Análise de Projetos de Pesquisa do Hospital das Clínicas da Universidade de São Paulo – CAPPesq/FMUSP), and written informed consent was obtained from the next of kin. From July 2010 to March 2011, in our 10 bed medical ICU (Hospital das Clínicas de São Paulo, Brazil), a convenience sample of 19 mechanically ventilated deeply sedated patients were enrolled and performed a single 20 minute passive cycling exercise.

**Figure 1:**
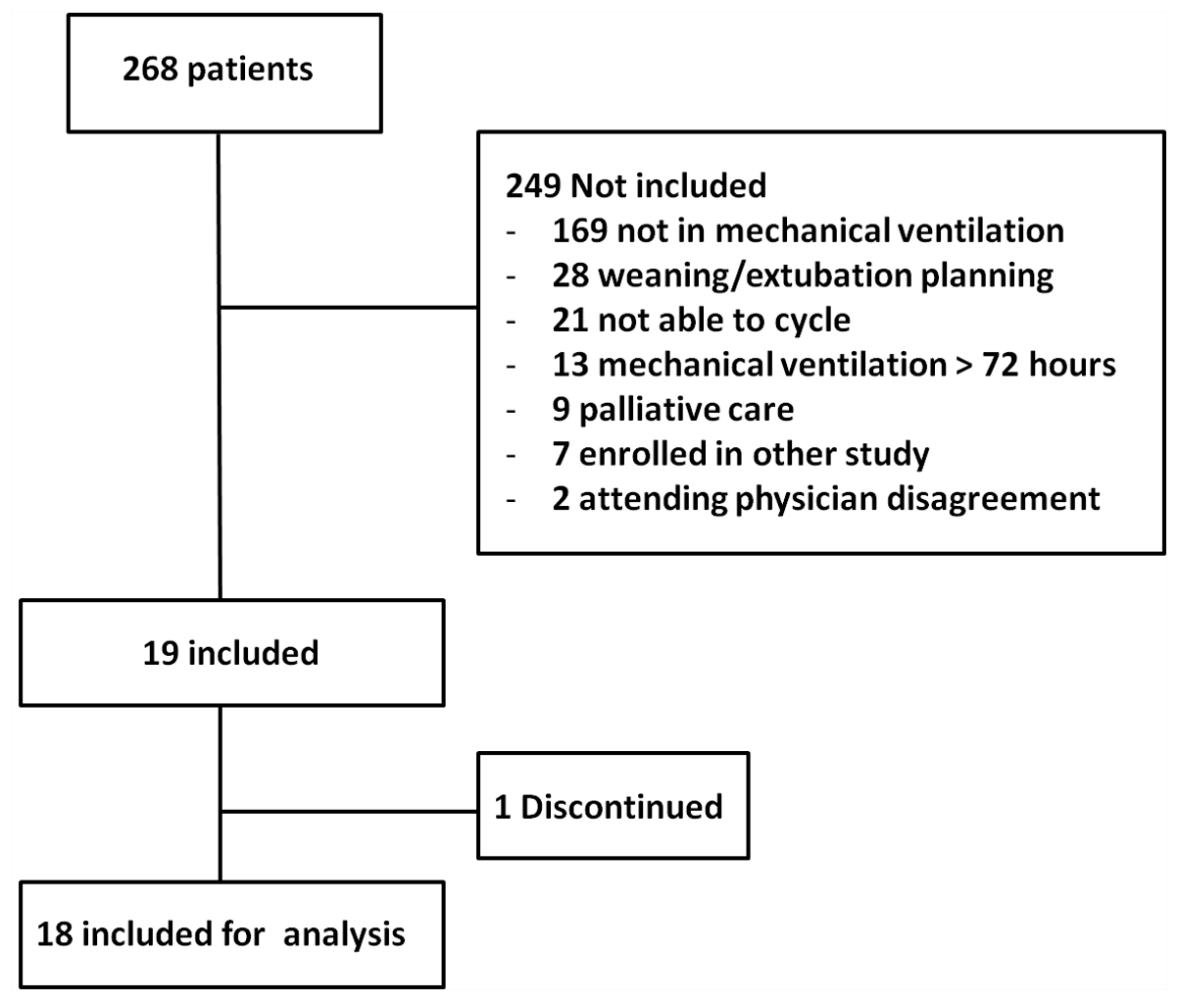
Flow chart of the protocol.

The inclusion criteria were mechanical ventilation <72 hrs, hemodynamic stability [mean arterial systemic pressure (MAP) >60 mmHg], absence of fever (<39 ^°^C), hemoglobin concentration >7 g/dL, a normal electrocardiogram in the previous hour and deep sedation level (SAS = 1) [7]. The exclusion criteria were as follows: lower limb problems that precluded exercise (for example leg bone tumor, deep vein thrombosis); enrollment in another research protocol; attending physician disagreement for enrollment; or patient under palliative care. The administration of a vasoactive agent was not an exclusion criterion, but changes in the infusion rate were not allowed during the protocol (Figure 1).

### Protocol

First, patients were maintained in bed and placed in the semi-recumbent position with their legs coupled to a cycle ergometer (Flexmotor – Cajumoro, São Paulo, Brazil). We then monitored the patient continuously for 35 mins, the duration of the protocol. Baseline variables (rest period) were recorded during the first five mins. A passive cycling exercise with a frequency of 30 revolutions per min was performed over the next 20 mins (supervised by the physiotherapist – RCPN, YMFK, ASH), and the variables were recorded minute-by-minute. After 20 mins, the exercise ceased and the recovery variables were recorded for the next ten minutes. Sedation (SAS = 1) was achieved with midazolam or propofol. Mechanical ventilation mode used was either pressure or volume controlled with a tidal volume of 6-8ml/kg (predicted body weight – GE-Engstrom Carestation, CT, USA). For all patients, fentanyl was used for analgesia. Sedative infusion rates and mechanical ventilation settings were not changed during the protocol.

The following criteria were used to discontinue the exercise: MAP <60 mmHg, systolic blood pressure (SBP) >200 mmHg, heart rate <40 beats/min and persistent peripheral arterial saturation <88%. Furthermore, the exercise was discontinued if any baseline parameter became deranged by more than 20% [2,4] and at the discretion of the attending physician.

### Hemodynamic, respiratory and metabolic

Analyzed parameters included minute-by-minute monitoring of cardiac output (CO), heart rate (HR), MAP, central venous pressure (CVP), systemic vascular resistance (SVR), peripheral oxygen saturation (SpO_2_), central venous oxygen saturation (ScvO_2_), respiratory rate (RR), tidal volume (Vt), oxygen consumption (VO_2_), carbon dioxide production (VCO_2_) and end tidal CO_2_ (ETCO_2_). Additionally, three blood samples (arterial and venous) for gas and lactate analyses were collected (at rest, exercise and recovery).

MAP was measured using an arterial catheter placed in the radial artery. Cardiac output was measured by the arterial waveform analysis (Flotrac-Vigileo System third generation - Edwards Lifesciences, CA, USA). This system calculates CO from the arterial pulse contour because stroke volume is physiologically related to arterial pressure, aortic compliance and arterial tone [8,9]. ScvO_2_ measurements were performed with a central venous oximetry catheter (Presep, Edward Lifesciences, CA, USA). This triple lumen catheter provides the means for solution infusion and CVP and SvcO_2_ monitoring by fiber optic reflectance spectrophotometry [10]. VO_2_, VCO_2_ and ETCO_2_ were measured using indirect calorimetry (GE-Engstrom Carestation, CT, USA) performed with a metabolic monitor designed for use in mechanically ventilated patients. This method has a fast differential paramagnetic O_2_ analyzer, an infrared analyzer for CO_2_, and a pneumotachograph to measure inspired and expired volumes [11].

Systemic vascular resistance was calculated as follows:

SVR = (MAP – CVP) x 80/ CO

Where SVR is systemic vascular resistance, MAP is mean arterial pressure, CVP is central venous pressure and CO is cardiac output. Values are expressed as dyne s/cm^5^.

Finally, during the exercise and for the next 48 hrs, we recorded any clinical adverse event that could have been associated with the exercise (new cardiac arrhythmias, hemodynamic instability or self extubation).

### Statistical analysis

Data distribution was analyzed with the Kolmogorov-Smirnov test. For each period (at rest, exercise and recovery) and patient, a single value of each parameter was calculated which represented the mean value of all the measurements in each period.

ANOVA for repeated measures with Bonferroni’s post test or the Friedman test with Dunn’s post test was undertaken according to data distribution using GraphPad Prism version 5.00 for Windows (GraphPad Software, San Diego CA USA). Data are presented as mean and standard deviation (SD) or median and 25-75% interquartile range (IQR) as appropriate. Changes in physiological parameters are expressed as mean, mean percentage of change and range of change (minimum and maximum values). The level of significance was set at *p* <.05.

## Results

Of 268 patients admitted, 19 patients (42% male, age 55±17 years) were enrolled (Figure 1). The demographic and clinical data of these patients are shown in Table 1. The median time of mechanical ventilation before enrollment was 1 day (0-2 days) with seven patients enrolled before 24 hrs. More than two-thirds (68%, n = 13) of our patients were administered vasoactive agents during the trial, and 21% (n = 4) required a norepinephrine dose ≥0.2 µg/kg/min (maximum dose = 0.47 µg/kg/min). One patient discontinued the exercise (15^th^ min) due to a painful condition related to undiagnosed bladder distension. The patient was treated accordingly and was excluded in the physiological analysis. Patients enrolled did not require modifications in mechanical ventilation settings, sedation and vasoactive drugs during the protocol and none of them were excluded due to attending physician discretion. None of the patients had rhabdomyolysis or had been administered neuromuscular blocking agents. The ICU mortality of the patients enrolled in the study was 21% (n=4).

**Table 1:** Demographic and clinical data of the patients.

**Characteristics**	**Value**
Male - n (%)	8 (42)
Age (years)	55 ± 17
Height (cm)	163 ± 10
SAPS 3 score	58 ± 13
SOFA (day of the protocol)	6 ± 3
Body Temperature (º C)	36.8 ± 0.8
ICU LOS (before enrollment - days)	1 (1-3)
MV (before enrollment – days)	1 (0 - 2)
ICU LOS (total - days)	10 (6-13)
MV (total – days)	4 (3-6)
Mechanical ventilation mode - n (%)	
Pressure controlled	17 (90)
Volume controlled	2 (10)
PEEP (cm H_2_O)	6 (5-8)
Driving Pressure (cm H_2_O)	14 ± 3
FIO_2_ (%)	42 ± 9
Respiratory rate - breaths / minute	23 ± 7
PaO_2_/FIO_2_	223 ± 75
Using norepinephrine - n (%)	13 (68)
Norepinephrine ≥ 0.2 µg.Kg^-1^.min^-1^ - n (%)	4 (21)
Diagnosis n (%)	
Pneumonia	10 (47)
ARDS	2 (10)
COPD	2 (10)
Chronic renal failure	2 (10)
Central nervous disorder	2 (10)
Asthma	1 (5)
Cause of MV n (%)	
Central nervous disorders	2 (10)
Sepsis/ Acute respiratory failure	17 (90)
ICU mortality (%) (n)	4 (21)
Blood gas analysis (baseline)	
Arterial pH	7.35 ± 0.09
PaO_2_ - torr	87 ± 19
PaCO_2_ - torr	44 ± 14
Arterial Lactate – mmol/L (baseline)	1.8 ± 0.6

Legend: SAPS – Scale for Assessment of Positive Symptoms; SOFA – Sequential Organ Failure Assessment; ICU – Intensive Care Unit; LOS – Length Of Stay; MV – Mechanical Ventilation; PEEP – Positive End Expiratory Pressure; FIO_2_ – Fraction of Inspired Oxygen; ARDS – Acute Respiratory Distress Syndrome; COPD – Chronic Obstructive Pulmonary Disease; PaO_2_ – Arterial Oxygen Partial Pressure; PaCO_2_ – Arterial Carbon Dioxide Partial Pressure.

### Respiratory outcomes

Compared with the rest values, RR and Vt (mean RR rest level = 23/min and mean Vt rest level = 350 mL) did not change during exercise and recovery.

### Hemodynamic outcomes

**Figure 2:**
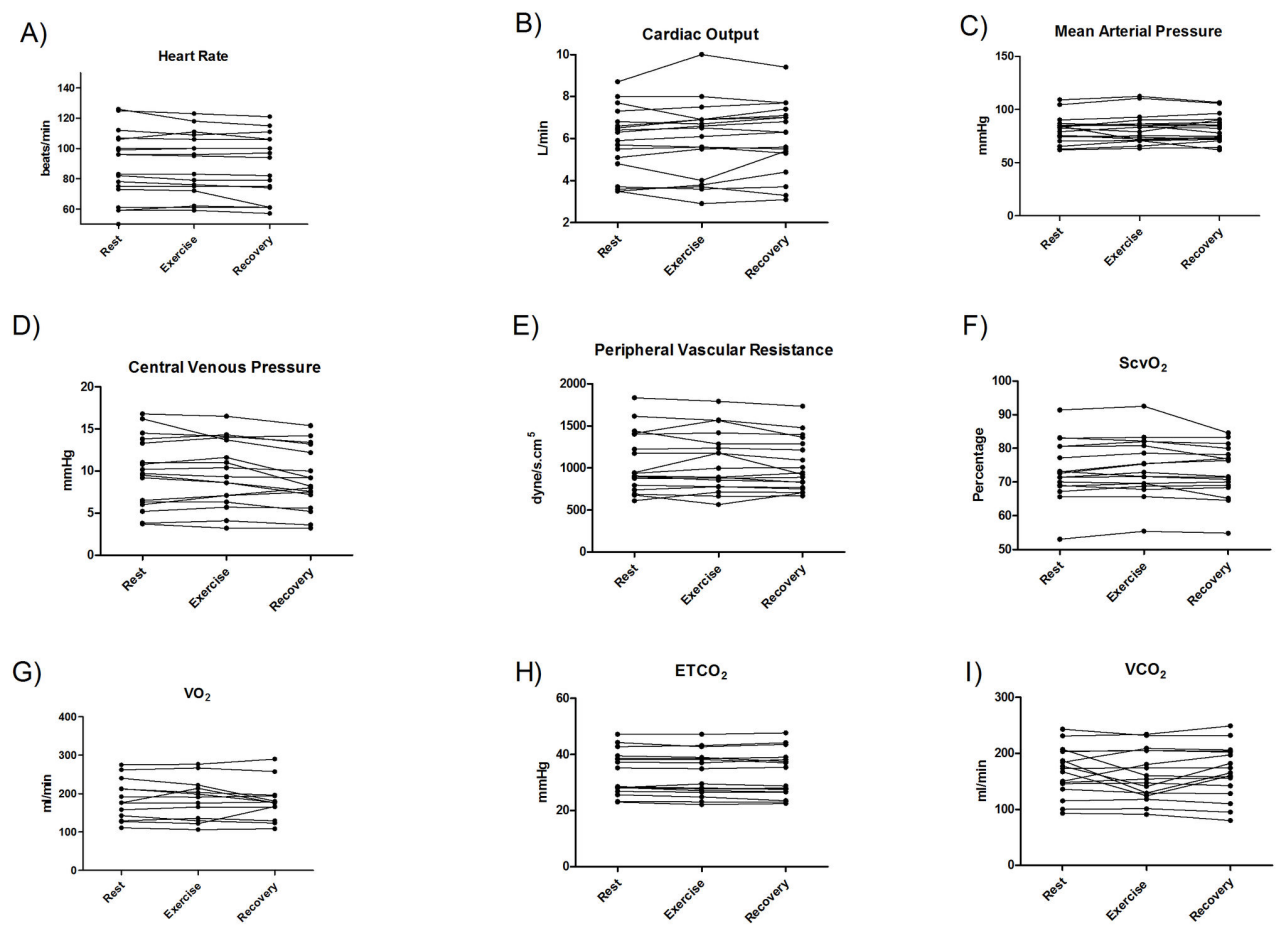
Hemodynamic and metabolic changes during the cycle ergometer exercise of the 18 enrolled patients. Legend: A-F) hemodynamic data; G-I) metabolic data. ScvO_2_ – central venous oxygen saturation; VO_2_ - oxygen consumption; VCO_2_ - carbon dioxide production; ETCO_2_ - end tidal CO_2_.

Compared with the rest values, there were no clinically relevant changes during the exercise in any of the hemodynamic variables. The HR rest value was 88 beats/min with a mean change of only 1% during exercise (-10% to 6%; NS) and 3% during recovery (-16% to 4%), the latter being statistically significant (*p* <.05). The CO rest value was 5.9 L/min with a mean change of 1% during the exercise (-16% to 15%) and no mean change during recovery (-9% to 27%) compared with rest. The MAP rest value was 81 mmHg with a mean change of 0.8% during exercise (-16% to 8%) and 0.6% after recovery (-13% to 13%; NS). The CVP rest value was 9.8 mmHg with a mean increase of only 1% during exercise (-15% to 18%; NS) and 7% after recovery (-25% to 25%), the latter being statistically significant (*p* <.05). The SVR rest value was 1059 dyne s/cm^5^ with a mean change of 0.8% during exercise (-17% to 25%) and 2.4% after recovery (-16% to 16%; NS). The ScvO_2_ rest value was 73.6% with a mean change of 0.9% during exercise (-2% to 4%) and 0.4% after recovery (-7% to 6%; NS) (Figure 2). The SatO_2_ rest value was 95% with no mean change during exercise (-4% to 1%) and recovery (-9% to 3%). Systolic blood pressure (SBP) rest value was 119 mmHg with a mean increase of 1.4% during exercise (-18% to 11%) and 3% after recovery (-18% to 20%; NS). The Diastolic blood pressure (DBP) rest value was 62 mmHg with a mean change of 0.6% during exercise (-14% to 8%) and 0.9% after recovery (-14% to 9%; NS).

### Metabolic outcomes

In accordance with the hemodynamic and respiratory outcomes, metabolic parameters did not change during the exercise and recovery when compared with rest values. VO_2_, VCO_2_ and ETCO_2_ analyses were available for 13, 17 and 18 patients, respectively. The VO_2_ rest value was 185.7 mL/min with no mean change during exercise (-9% to 21%) and 2% after recovery (-25% to 31%; NS). The VCO_2_ rest value was 168.5 mL/min with a mean increase of 5% during exercise (-30% to 21%) and 1% after recovery (-24% to 31%; NS). The ETCO_2_ rest value was 32.8 mmHg with a mean change of 0.9% during exercise (-4% to 5%) and after recovery (-8% to 3%; NS) (Figure 2).

Additionally, we did not find any statistically significant difference in pH (7.35, 7.36 and 7.35), PaO_2_ (87, 91 and 93 torr), PaCO_2_ (44, 43 and 43 torr) and lactate levels (1.8, 1.6 and 1.6 mmol/L) when comparing rest, exercise and recovery measurements, respectively.

Finally, we analyzed three subgroups of patients [norepinephrine ≥0.2 µg/kg/min (n = 4), PaO_2_/FIO_2_ <150(n = 4) and ScvO_2_ <70%(n = 8)] and the results were similar to those of the overall population.

### Adverse events

Two patients had adverse events related to the exercise. In one patient, there was an increase in respiratory frequency caused by auto-triggering of the ventilator when the exercise started. The patient was obese, and we hypothesized that the cause of the auto-triggering was an increase in abdominal pressure due to the cycling movement. In this patient, we decreased the trigger sensitivity of the ventilator, and the problem resolved. In the other adverse effect, the patient awoke in the fifteenth minute and resisted the cycling action. In this case, we stopped the exercise and requested the attending physician to evaluate the patient. Bladder distention was diagnosed and treated accordingly. After this, the patient was allowed to rest and excluded from the analysis. The patient had no other adverse event related to the protocol during ICU stay. Including this patient into the analysis (rest x exercise) the results were similar than described above.

In the following 48 hrs after the exercise, we observed no hemodynamic, respiratory or metabolic adverse effects that could be related to the exercise. Additionally, we did not report any muscle or skeletal adverse event related to cycling activity in our patients.

## Discussion

We observed that a very early cycling exercise is feasible and can be performed safely for mechanically ventilated patients in the ICU. The exercise did not significantly change the patients’ hemodynamic, respiratory and metabolic rest parameters, even those receiving high doses of norepinephrine and those with a low oxygen index or ScvO_2_ <70%. Although we observed a significant difference in HR and CVP, these small differences were observed when comparing rest with the recovery period and are not clinically relevant.

In 2007, Bailey and colleagues first reported that patients in critical care units could walk, even when mechanically ventilated. After six days of ICU admission they sat the patients on the edge of the bed [1]. Since then, several studies have shown that early activity could enhance recovery [12,13] even when very early protocols (within 1-3 days of mechanical ventilation) are used [3,4]. Finally, Burtin and colleagues reported that critical care patients could cycle; however, they started the intervention after 14 days of ICU admission [6]. To the best of our knowledge, our study is one of the earliest mobilization therapy interventions reported in critically ill patients, when compared with those previously published [1,3,4,6,13]. Morris and colleagues began their protocol within 48 hrs of mechanical ventilation [3], and Schweickert and colleagues started theirs after 24 hours of mechanical ventilation [4]. Indeed, 35% of our patients undertook the cycling trial after less than 24 hrs of mechanical ventilation with no adverse effects.

According to the European Respiratory Society and European Society of Intensive Care Medicine, passive and active exercises should be commenced as soon as possible in critically ill patients [14]. Passive motion can be defined as a repeated movement of a joint within its normal range [15]. Although this technique is widely used in ICU patients [16–18], there is a lack of data regarding its benefits. Theoretically, it may maintain and recover range of movement, decrease synovial fluid stasis by producing fluctuations in intra-articular pressure [15], prevent contracture and promote function [14]. We choose to study passive exercise for the following reasons: 1) deep sedation is still indicated for a limited period of mechanical ventilation [19], and although numerous studies have shown that maintaining deep sedation is associated with worse outcomes, this is still employed in most ICUs around the world [19,20] and in this context passive mobilization is the only feasible type of exercise; and 2) a less sedated patient could interfere with the exercise by assisting the cycling movement. In the latter circumstance, we would not have been able to establish whether any hemodynamic or metabolic changes observed during the exercise were caused by passive movement or the patient’s own efforts.

Few studies have evaluated hemodynamic and metabolic changes following physiotherapy treatment in the ICU. Burtin and colleagues found no general changes in HR, SBP and DBP, although SpO_2_ decreased by 1%. Exercise was interrupted in six sessions (out of an overall 425) due to an increase of SBP >180 mmHg [6]. However, these investigators performed either passive or active cycling exercises, and we believe that hypertension occurred during active exercise (although this was not reported by the authors). Norremberg and colleagues [21] reported increases in CO and VO_2_ in 16 ICU patients after passive leg movement. However, in that study, most of the patients were not sedated and we cannot be certain that muscle activation occurred even though participants were asked to stay calm during the mobilization. In our study, all the patients were sedated which attenuates hemodynamic and metabolic responses during ICU procedures [22]. Furthermore, it can be argued that the range of motion applied in our study was not sufficient to stretch the muscle or generate a passive muscle pump to increase venous return or activate muscle mechanoreflexes [21,23].

Some limitations of our study should be acknowledged. First, we used the FloTrac-Vigileo system to measure CO. Although this technique is validated for patients with sepsis [8], it may not accurately track changes in cardiac index induced by volume expansion or changes in norepinephrine dose [24] that can alter vascular tone and compliance. In our study, changes in the doses of vasoactive agents and volume expansion were not allowed during the protocol, and patients were observed for at least one hour before enrollment. Second, we do not have metabolic data available for all patients, and some data were not recorded during the protocol. This measurement technique also has some limitations under circumstances of high FIO_2,_ high respiratory rate, leaks around the endotracheal tube cuff, and obstruction of the sensor line by water vapor [25]. Nevertheless, we did not observe any alteration in PO_2_ and PCO_2_ in blood gas analyses (arterial and venous), and together with an unchanged CO, we did not expect any change in metabolic outcome measures. Third, though we have verified that the passive cycling exercise is feasible and safe, our study was not designed to examine the benefits of this very early intervention in critically ill patients.

We showed that in our population a very early passive cycling exercise in sedated, critically ill, mechanically ventilated patients is feasible and safe. Early passive cycling is not associated with significant alterations in hemodynamic, respiratory or metabolic variables. This very early mobilization might be associated with better outcomes in the ICU survivals, especially to joints and muscles. However, this hypothesis needs to be confirmed in future clinical studies.
